# Application of imaging mass cytometry for spatially profiling the microenvironment of salivary glands in primary Sjögren’s syndrome

**DOI:** 10.1038/s41419-025-07717-7

**Published:** 2025-05-16

**Authors:** Guolin Wu, Fangping Wu, Lipei Wang, Lixiong Ying, Wenwen Lu, Kang Qian, Tianxiao Fu, Danbin Wu, Fenglin Hu, YiHang Shi, Li Xu

**Affiliations:** 1 Department of Traditional Chinese Medicine, The First Affiliated Hospital, Zhejiang University School of Medicine, Hangzhou, Zhejiang China; 2 School of Pharmaceutical Sciences, Zhejiang Chinese Medical University, Hangzhou, Zhejiang China; 3 Hangzhou Normal University of Basic Medical Sciences, Hangzhou, Zhejiang China; 4 The First Affiliated Hospital, Zhejiang University School of Medicine, Hangzhou, Zhejiang China

**Keywords:** Metabolic syndrome, Immunology

## Abstract

Primary Sjogren’s syndrome (pSS) is a slowly progressive, systemic autoimmune disorder characterized by gradual lymphocytic infiltration of exocrine glands. However, the spatially profiling the immune microenvironment in pSS is largely unclear, limiting the understanding of the complex interplay among cells within the microenvironment. Based on imaging mass cytometry (IMC) analysis of clinical pSS samples, we first revealed that labial salivary gland (LSG) comprised of epithelial, immune cells and stromal cells, and epithelial was the main cell type in LSG. Eight immune cells populations were identified, including CD8^+^ T, CD4^+^ T, Treg, B, NK cells, neutrophils, resident macrophages and a mixed immune cell cluster. We found that CD8^+^ T cells, but not CD4^+^ T cells, were the most prominent T cells in immune infiltrates of pSS LSG. With the increase of pSS disease activity and severity, the infiltration abundance of CD8^+^ T cells gradually increased and was accompanied by the activation of inflammatory response. sc-RNA-seq analysis based on the GSE272409 dataset confirmed that CD8^+^ T cells were the main immune cells, and dominated the most intercellular ligand-receptor interactions. CD8^+^ T cells were further clustered into five cell subsets, of which CD160^+^CD8^+^ T cells subset appeared to present only in pSS patients. Further experiments demonstrated that CD160 expression on CD8^+^ T cells was associated with an enhanced expression of proinflammatory and cytotoxic cytokines IFN-γ, GZMB and TNF-α, and the injury of salivary gland epithelial cells. Besides, proportion of GZMK^+^CD8^+^ T cells subset was increased in pSS patients. Trajectory analysis confirmed an enhanced frequency of CD8^+^ T cell differentiation and activation during the progression of pSS. This study provided single cell profile with spatial information for analyzing the LSG immune microenvironment in pSS, which could not be achieved by conventional immunofluorescence and immunohistochemistry assays.

## Highlights

- Our IMC analysis identified CD8^+^ T cells as the most prominent T cells in immune infiltrates in labial salivary gland of pSS.
- Epithelial and their interactions with immune cells may play important roles in the treatment response in pSS.
- CD160^+^CD8^+^ T cells subset presented only in pSS patients, but not controls.
- CD8^+^ T cell differentiation and activation were more frequent during the progression of pSS.
- CD160^+^CD8^+^ T cells may exert a proinflammatory and cytotoxic role in pSS pathogenesis.

## Introduction

Sjögren’s syndrome is a chronic systemic autoimmune disorder characterized by the gradual lymphocytic infiltration of exocrine glands (mainly salivary and lacrimal glands), which causes decreased function of exocrine glands, thus resulting in xerophthalmia and xerostomia [1]. Sjögren’s syndrome can be divided into primary or secondary these two forms. Primary Sjögren’s syndrome (pSS) has become increasingly common with a prevalence ranging from 0.3% to 3% among the general population [2]. The incidence of pSS varies across countries and regions, and differences in diagnosis may be the one for interpretating such differences on incidence. There is no single “gold standard” for diagnosis of pSS, and the diagnosis of this disease mainly relies on a combination of tests, including symptom assessment, laboratory tests, and salivary gland biopsy [3]. Although the current ACR-EULAR classification criteria have certain sensitivity and specificity, there are still some early or atypical cases that cannot be accurately identified in practical application [4]. Besides, the symptoms of pSS patients are diverse and lack specificity, and symptoms such as dry mouth and dry eyes may overlap with other diseases, causing difficulties in diagnosis. In addition to dryness, one third of patients have systemic involvement [5], involving skin, thyroid, renal, lung, and even the nervous systems [6–9].

Lacking of illustration on the pathologic mechanisms of pSS causes an unmet therapeutic for this disease. Specifically, current treatment for pSS primarily focuses on symptomatic relief. For example, artificial tears and saliva substitutes can relieve the symptoms of dry mouth and dry eyes, but they cannot be cured. Nonsteroidal anti-inflammatory drugs, glucocorticoids and immunosuppressants such as methotrexate and mycophenolate [10], can alleviate immune inflammation, but long-term use leads to greater side effects, and the efficacy varies greatly among patients. Besides, the manifestations of different symptoms and organs should be taken into accounts in the treatments due to the multiple systems involvements [11, 12]. Such treatment strategies are largely empirical rather than are formed based on evidence [5]. Therefore, illustrating the abnormal activation mechanism of the immune system in the context of pSS is indispensable to enable a more targeted therapeutic approach.

Increasing evidences have demonstrated the pathogenic role of the immune microenvironment in mediating pSS progression [13–15]. For example, Christodoulou et al. [14] revealed that immune infiltrate composition at the minor salivary gland lesions of pSS varied based on lesion severity and showed correlations with the disease manifestations. T, B and natural killer (NK) lymphocytes comprises the persistent inflammatory lesions of pSS, and the accumulation of T cells tends to occur in the early stage, while B cells in later stage [16]. However, such characterization of immune microenvironment in exocrine glands are investigated largely depending on nonquantitative and low dimensional histological staining [17–19], which leads to an inadequate understanding of etiopathogenesis.

Recently, RNA-Sequencing (RNA-seq) combined with the computational approaches have contributed to elucidate the pSS microenvironment by estimating immune components according to well-established immune-specific marker gene sets [20, 21]. RNA-seq at the single-cell level further facilitates our understanding of the cellular heterogeneity and the complexity of changes among cell types or subsets [22, 23]. Besides, a previous study identified a 6-cell signature as biomarker for stratifying pSS patients by using flow cytometry time-of-flight (CyTOF) profiling of peripheral blood mononuclear cells [24]. Nevertheless, a key limitation of both single-cell sequencing and CyTOF is their incapacity for capturing tissue architecture and spatial information because that both these two approaches rely on dissociated cells.

Imaging mass cytometry (IMC) is a novo technology enables to study the spatial and phenotypic information of disease by simultaneous analysis of multiple protein targets in tissue sections stained with metal-tagged antibodies at single-cell resolution [25]. Herein, we used IMC to spatially quantify 39 markers at subcellular resolution in labial salivary gland (LSG) samples of 15 pSS patients and 3 normal controls, which enable us to comprehensively characterize the cells composition and spatial communications within pSS microenvironment. We also compared the microenvironment heterogeneity among different pSS subgroups, and the correlations with treatment response. Overall, this study provides a comprehensive understanding on the immune microenvironment underlying pSS development, and sheds a light on the treatment strategies for pSS.

## Materials and methods

### Human subjects and samples collection

For all the patients, written informed consents were obtained, and the protocol was approved by the Ethics Committee of the first affiliated hospital of Zhejiang University School of Medicine (IIT20240145B). Eighteen formalin-fixed paraffin-embedded (FFPE) LSG biopsy samples derived from individuals for clinical treatment were obtained from our hospital. Individuals were included: those aged 18–75 years and received antibodies examination in sera, eye examination and LSG biopsy, and signed informed consent. Individuals met the following criteria were exclude: 1) severe cardiopulmonary insufficiency; 2) suffering from liver, kidney, hematopoietic system and other serious primary diseases; and 3) those combined with other rheumatic immune system diseases and received hormones or immunosuppressive treatment. Patients with pSS were diagnosed based on the 2016 American College of Rheumatology (ACR)/EULAR criteria [26], and those with the EULAR Sjögren’s syndrome disease activity index (ESSDAI) ≥ 4 were confirmed with pSS (*n* = 15). Whereas those with ESSDAI < 4 were considered to have no pSS and were regarded as healthy controls (*n* = 3). The pSS patients were further stratified according to SSA antibody in sera and pathological hematoxylin-eosin (H&E) staining in lesions, including positive for HE staining and negative for SSA antibody (HE^+^, SSA^–^, *n* = 5), negative for HE staining and positive for SSA antibody (HE^–^, SSA^+^, *n* = 5) and positive for both HE staining and SSA antibody (HE^+^, SSA^+^, *n* = 5). Clinical data of all these 18 individuals were retrospectively reviewed, and the detailed information for each were listed in Table S1. All pSS patients were given conventional treatment with hydroxychloroquine sulfate tablets (Shanghai Zhongxi Pharmaceutical Co., LTD., China), 0.2 g each time, orally, twice a day. For those with obvious joint pain, they received oral diclofenac sodium enteric-coated tablet (Beijing Novartis Pharmaceutical Co., LTD., China), 75 mg once a day. For those with obvious dry eyes, sodium hyaluronate eye drops (Shentian Pharmaceutical Co., LTD., China) was used with one drop for each eye, four times a day. The course of treatment was 24 weeks, and the condition was evaluated again. After treatments, whose ESSDA score fell below 4 to be considered responsive to treatment (response, ESSDA < 4 after treatment), while those whose ESSDA score remained greater than 4 were considered unresponsive to treatment (non-response, ESSDA ≥ 4 after treatment).

### IMC sample preparation and acquisition

A representative H&E stained slide was prepared for each sample and reviewed by a dedicated dermatopathologist at our hosptial to determine a region of interest (ROI), which was then mapped to the FFPE tissue blocks for further IMC. Briefly, FFPE tissue slides were heated for 1 h at 68 °C, dewaxed in xylene twice, followed by rehydration orderly in 95%, 85%, and 75% ethanol for 5 min each. Antigen retrieval was then performed by placing the slides into citric acid buffer and heating at 100 °C for 30 min. After cooling, the slides were washed with ddH_2_O and PBS for 10 min each, and then were blocked by SuperBlock for 30 min. Next, the slides were incubated with metal-tagged antibodies, and the antibody panel was listed in Table S2. The stained tissue slides were scanned by means of imaging mass cytometer (Fluidigm, Hyperion) to generate the multiplexed images.

### IMC image preprocessing and cell segmentation pipeline

There involved several steps across the IMC image preprocessing. First, the spillover signal in each channel was filtered by the method described in a previous study [27]. Then, median filtering was utilized for image denoise. Next, to enhance the contrast between the real signal and the background, the linear adjustment method is used to adjust the intensity. Finally, cell segmentation on the IMC images was performed utilizing the pre-trained TissueNet, which was introduced in a previous work [28]. The TissueNet requires two channels of imaging data for its operation. The first channel corresponds to the nuclear channel, typically labeled with DAPI, which provides information about the locations of cell nuclei. The second channel represents either the membrane or cytoplasmic channel, indicating the cell boundaries. In our study, we specifically chose the membrane channels to serve as the second channel.

### IMC data analysis

Expression of markers was range normalized to the 99th percentile across all cells for each channel separately and batch effects was then aligned by means of R package Harmony (version 0.1.0). Next, cells clustering analysis was conducted by means of the R package Rphenograph (version 0.99.1) [29], and dimensionality reduction was conducted utilizing UMAP method to visualize the distribution of cell clusters at a single cell level. For cellular neighborhood (CN) analysis, the nearest 20 neighboring cells were considered to consist of the CN for each cell in this study. These neighborhoods were grouped and annotated according to their cell components using k-means clustering method (*k* = 10), and the results were verified by superimposing Voronoi plots of corresponding cell neighborhoods on the original tissue IMC image. K-means is a distance-based clustering algorithm. Similarity between two observations or samples is usually measured by distance in the algorithm. The Euclidean distance [30, 31] was selected for the K-means algorithm in this study.

### Spatial analysis

The cell-cell spatial interactions counting was defined as the number of each cell type within a CN of a certain cell. The permutation test in imcRtools (version 1.0.2) [32] was employed to determine whether there was a statistically significant interactions/avoidances between each cell type within each CN in comparison with that of random observations. Permutation test is a nonparametric statistical method based on data rearrangement (permutation) that is used to test the null hypothesis in a hypothesis test [33]. It does not depend on the distribution assumptions of the data and can be used for various statistics and complex experimental designs. It is particularly valuable when few independent observations are available. Student’s *t* test was used to analyze the difference on the interactions between different groups. Student’s *t* test is a commonly used statistical test, mainly used to test whether two independent samples come from a population with the same mean. It is based on a t distribution and is suitable for situations where the sample size is small (usually less than 30) and the data follows a normal distribution. *P* < 0.05 was considered as significant difference in spatial interaction between different groups.

### Single-cell RNA sequencing (sc-RNA-seq) data analysis

The sc-RNA-seq for pSS (GSE272409) was downloaded from GEO database, which contains LSG samples derived from 7 pSS cases and 6 normal controls. Quality control of the scRNA-seq data was conducted by means of “Seurat” package (version 4.4.0). Specifically, cells expressing genes fewer than 200 genes, cells with over 10% mitochondrial gene expression, and cells with over 3% erythrocyte gene expression were excluded. Finally, 30,964 appropriate cells and 23,074 genes were identified. The top 2000 highly variable genes were identified by means of “FindVariableFeatures” function. The “ScaleData” function was employed to appropriately expand the data, and the top 30 principal components were utilized for subsequent analyses. The cell clusters were identified utilizing “FindNeighbors” and “FindClusters” functions (resolution = 0.5). The dimensionality reduction and visualization of the cell culsters was conducted utilizing UPAP method. Cell clusters were annotated based on the markers provided in CellMark 2.0 website. Genes differentially expressed among cell clusters were analyzed utilizing “FindAllMarkers” function with the cut-off values of |log2FC | > 0.2 and *P* < 0.05. Gene involved gene ontology and KEGG pathways were explored by enrichment analysis, which was conducted by clusterProfiler package (version 4.10.1). The monocle2 package (version 3.20.1) and the CellChat package (version 1.6.1) were utilized for Pseudotime analysis and intercellular communication, respectively.

### Animals

NOD mouse is a commonly used model for pSS because that it spontaneously develops human pSS-like symptoms, including lymphocytic infiltration in the salivary glands, and autoantibodies [34–36]. Female NOD/Ltj mice (12 weeks) were used as pSS animal model, and sex and age matched outbred ICR mice were used as controls. All mice were fed in a SPF environment with free access for water and food, and the room temperature was controlled at 25 °C with a 12:12-h light/dark cycle. Whole experiments were supported by the Animal Experimental Ethics Committee of Yangzhou University (Ethics No. 202504019), which were conducted in accordance with ARRIVE guidelines.

### Flow cytometry (FCM)

The mice were sacrificed by cervical dislocation under deep anesthesia (intraperitoneal injection of 50 mg/kg sodium pentobarbital), and the SG were isolated immediately. The isolated SG were cut into small pieces, and then were prepared into cell suspension after digestion using Hank’s solution. After washing, the cells were resuspended in MojoSort™ Buffer, and the suspension was filtered in a 70 μm Cell Strainers. The obtained cells were collected, and were analyzed by FCM after stained by anti-CD45 (#157213, Biolegend, USA) and anti-CD160 (#143003, Biolegend, USA). Besides, CD45^+^ cells were further isolated from the cell suspension by magnetic-activated cell separation (MASC) using MojoSort™ Mouse CD45 Nanobeads (#480027, Biolegend, USA), and then were analyzed by FCM after stained by anti-CD8 (#100705, Biolegend, USA) and anti-CD160 antibodies (#143003, Biolegend, USA). All analyses were performed using an CytoFLEX S Flow Cytometer (Beckman, USA) and interpreted using the FlowJo software.

### Immunofluorescence

SG tissues were fixed in 4% paraformaldehyde and embedded by Frozen Section Embedding Medium, and were prepared into sections for immunofluorescence staining. For staining, sections were rewarmed, then were soaked and washed with PBS for three times. After blocking by 5% BSA, primary antibodies (anti-CD8 and anti-CD160) were incubated overnight at 4 °C, followed by incubation with secondary antibodies and DAPI staining in turn. After drying, the sections were sealed with fluorescent quenching sealant-contained sealing solution, and were examined under a confocal laser scanning microscopy.

### Primary culture of salivary gland epithelial cells (SGECs)

The isolated SG samples from the ICR mice (controls) was incubated with type II collagenase (0.5 mg/mL) and DNA enzyme I (0.1 mg/mL) at 37 °C for 1.5 h. The digested tissue was then processed into cell suspensions after filtration (70 μm) and erythrocyte lysis. Afterwards, cells were resuspended and cultured in the complete medium (F12 DMEM of 3:1, 2.5% FBS, 10 μg/L epidermal growth factor, 2 mmol/L L-glutamine, 100 U/mL penicillin, 100 mg/L streptomycin).

### Isolation of CD8^+^CD160^+^ T cells and coculturing with SGECs

CD8^+^CD160^+^ T cells in SG of the NOD/Ltj mice were isolated using MASC. Briefly, SG samples were processed into cell suspensions via mechanical dissociation, filtration (70 μm), and erythrocyte lysis as described above. CD8^+^ T cells were then isolated with the aids of a mouse CD8^+^ T cell isolation kit (#B90011, Selleck) as per the manufacturer’s manual. Next, CD8^+^ T cells were incubated with phycoerythrin (PE)-anti-CD160 and then separated into CD160^−^ and CD160^+^ subsets using PE-Positive Selection kit (Stemcell). The obtained CD8^+^CD160^+^ T cells were cultured in in RPMI 1640 medium with 10% FBS, 2 mM L-glutamine, 100 U/ml penicillin, and 100 mg/ml streptomycin, and were activated by T-Activator CD3 (2 µg/mL) and CD28 (1 µg/mL). For coculturing, CD8^+^CD160^+^ T cells (1 × 10^6^) and SGECs (5 × 10^5^) were added to the upper (with 0.4 µm membrane pore) and lower chamber of Transwell, respectively. Following 24 h of incubation, cells were collected for cytotoxicity assays.

### Cytotoxicity assays

Cytotoxicity of CD8^+^CD160^+^ T cells to SGECs were examined by determining cell proliferation and apoptosis. For proliferation, 10 μL of CCK-8 solution was added for each well for another 2 h of culture, and absorbance at 450 nm was measured to evaluate the cells viability. For apoptosis, cells were collected and resuspended in binding buffer. After staining by Annexin V-FITC and propidium iodide, cell apoptosis was determined by FCM.

### Cell stimulation and RT-qPCR

RT-qPCR was used for detecting the expression of markers (IFN-γ, GZMB and TNF-α) of CD8^+^CD160^+^ and CD8^+^CD160^−^ subsets. Cells were stimulated with Phorbol myristate acetate (PMA) (50 ng/mL) + ionomycin (750 ng/mL) for 4 h at 37 °C, and then cells were collected for analysis. Briefly, total RNA was extracted utilizing TRIzol reagent, followed by the synthesis of cDNA through reverse transcription. RT-qPCR was performed using SYBR Green PCR Master Mix. The used primers were listed in Table S3.

### Statistical analysis

All data analysis was conducted by R (version 4.3.3). Wilcoxon rank sum test was employed for comparisons between two groups in scRNA-seq analysis. Wilcoxon rank sum test is a nonparametric statistical test used to compare the medians of two independent samples for significant differences. This method is based on sorting the two groups of data after merging, and then calculating the rank sum of the two groups of data, and judging whether the difference between the two groups of data is significant by comparing the rank sum. Correlations were evaluated by Sperman correlation analysis. All experimental data was presented as mean ± standard deviation, and were statistically graphed by GraphPad 7.0 software. Unpaired t-test, also known as an independent sample t-test, is used to compare the mean/average of two independent or unrelated groups to determine if there is a significant difference between the two. In this study, comparison for experimental data between two groups was conducted by unpaired t-test, with *P* < 0.05 representing statistically significant.

## Results

### A multiplexed antibody panel was developed to characterize the microenvironment of pSS

**Fig. 1:**
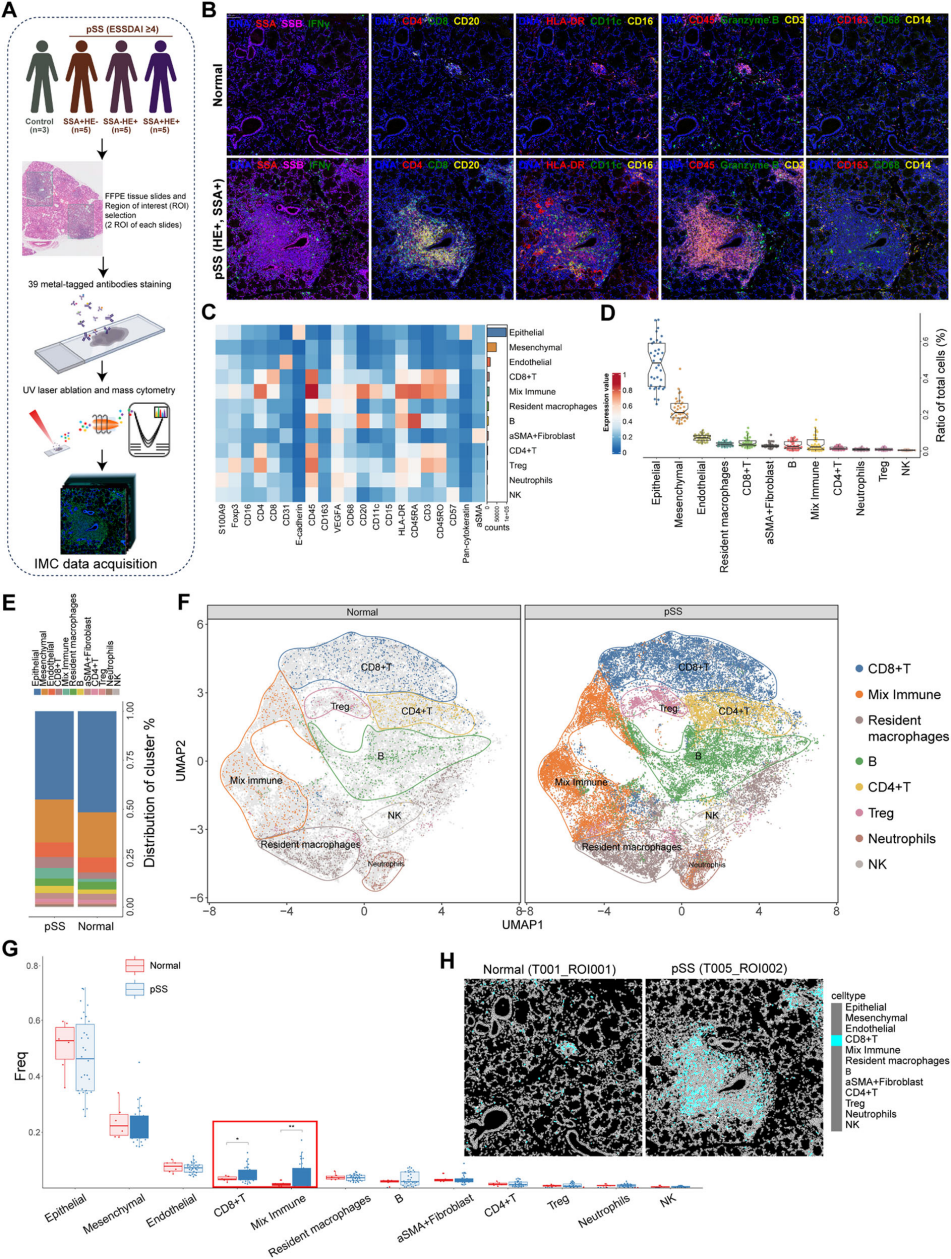
Characterization of the pSS microenvironment. **A** The study design of this imaging mass cytometry study. We performed imaging mass cytometry (IMC) analysis using a 39-antibody panel on 36 regions of interest of labial salivary gland biopsy samples across 15 pSS patients and 3 controls. After preprocessing and cell segmentation, IMC data was acquired for downstream analyses; **B** Multiplexed images showing the staining of cellular markers (pSS pathological markers and major lymphocyte markers) in labial salivary gland samples from normal control and pSS patients; **C** Heatmap demonstrating the expression of cellular markers in each cell population; **D** Boxplots showing the ratio of each cell type of the total cells in all samples; **E** Histogram demonstrating the differences on cell proportion in normal control and pSS patients; **F** the UMAP clustering of immune cells in normal control and pSS patients; **G** Comparisons on the cells frequency between normal control and pSS patients, *P < 0.05 and **P < 0.01; **H** the representative cell Voronoi map showing the distribution of CD8^+^ T cells in ROI of normal control and pSS patient.

To extensively characterize the microenvironment of pSS, a multiplexed antibody panel (39 markers and DNA) was developed for application on LSG biopsy samples, which was analyzed as the workflow in Fig. 1A. Our IMC panel covered markers for delineating various immune cells infiltration, epithelial, endothelial, fibroblast, and mesenchymal cells, along with pSS markers (Fig. S1). Compared to healthy control, the LSG biopsy samples of pSS patients exhibited an overall enhanced lymphocyte and inflammatory cells infiltration (Fig. 1B). We further visualized the cell clusters with the aids of UMAP dimensionality reduction tool. Overall, based on the expression patterns of markers, 12 cell populations were definitively identified and annotated, including 4 structural cells (such as epithelial and mesenchymal cells) and 8 immune cells (Fig. 1C and Fig. S2A). There was an obviously higher proportion of structural cells than immune cells (Fig. 1D and Fig. S2A). To make a more clearly visualization on infiltrating immune cells, we firstly separated all cells into structural cells and immune cells, followed by UMAP clustering (Fig. S2B-C). The main cell type of structural cells was the epithelial cells, followed by mesenchymal cells, endothelial cells and α-SMA^+^ fibroblast (Fig. S3A). The infiltrated immune cells included CD8^+^ T, CD4^+^ T, regulatory T (Treg), B, NK cells, neutrophils and resident macrophages. Of these immune cells, resident macrophages, CD8^+^ T, and B cells were the main cell types showing higher percentage, and the high percentage of mixed immune cells indicated the close interactions among these immune cells (Fig. S3B). These results indicated that the microenvironment of pSS comprised of epithelial, mesenchymal, endothelial and immune cells. Compared to healthy control, the proportion of epithelial cells decreased, while the proportion of α-SMA^+^ fibroblast, mixed immune cells, B cells and CD4^+^ T cells were elevated in pSS patients (Fig. 1E). Although all immune cell populations were present in both healthy and pSS samples, there were significantly higher fractions in cell numbers per cluster in pSS samples, indicating that there were significant immune cells infiltration in pSS (Fig. 1F). Differential analysis indicated that pSS sample harbored significant higher proportion of mixed immune cells, particularly CD8^+^ T cells (Fig. 1G, H).

### Infiltration levels of CD8^+^ T cells correlated with disease severity and inflammatory

**Fig. 2:**
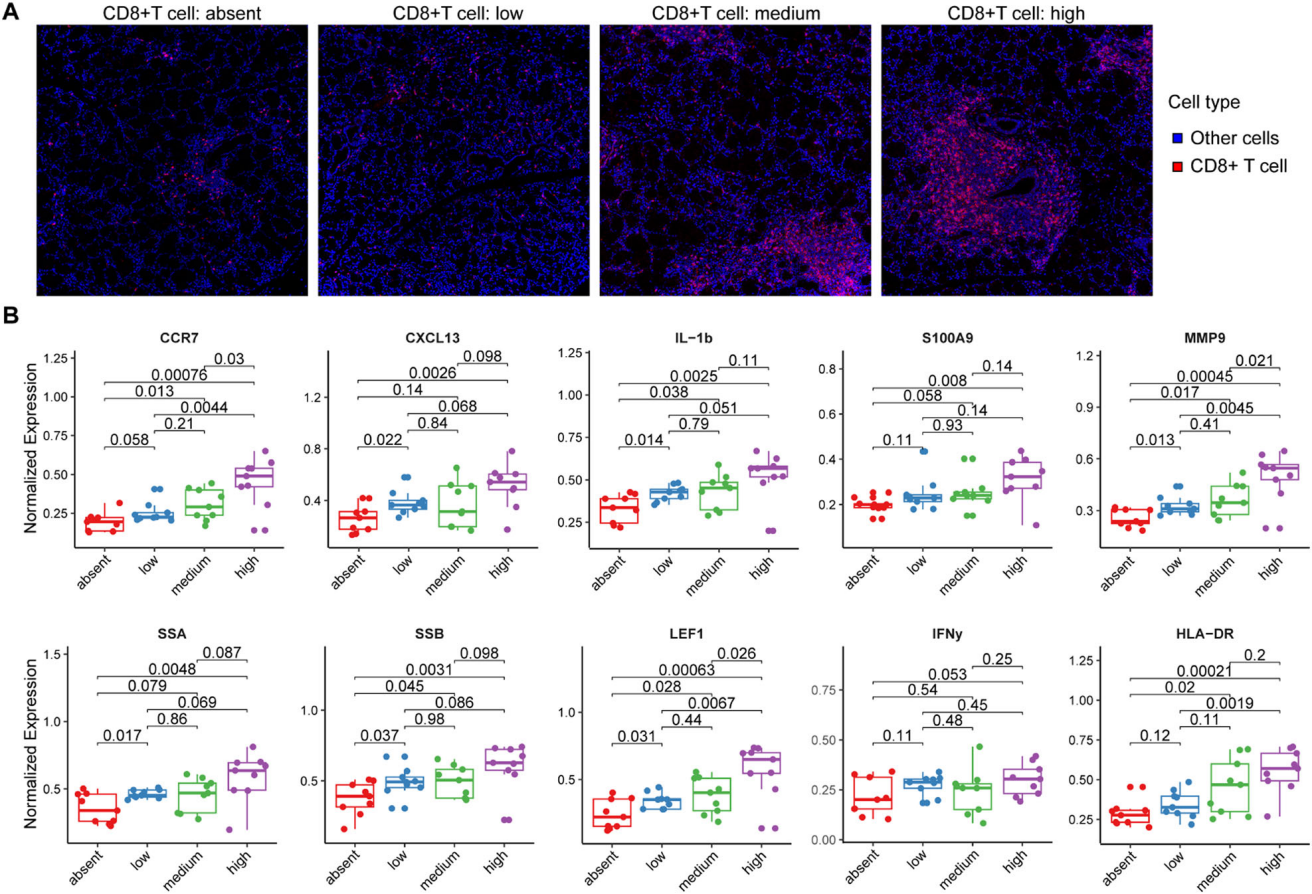
Associations of CD8^+^ T cells with disease progression and inflammatory. **A** the representative images showing different density of CD8^+^ T cells infiltration. To investigate the associations of CD8^+^ T cells with disease progression and inflammatory, we formed four equally sized groups (absent, low, medium and high) of images ranging from CD8^+^ T cell absence to high infiltration; **B** box plots showing the expression of functional factors (pSS pathological markers and inflammatory cytokines) in four groups divided by the CD8^+^ T cell infiltration: absent, low, medium and high CD8^+^ T cell infiltration in their LSG.

Considering the prominent manifestation of CD8^+^ T cells in above analysis, we proposed that CD8^+^ T cells might play an important role in the occurrence and development of pSS. Hence, we focused more deeply on the association of functional factors-expressing cells with CD8^+^ T cell infiltration. We firstly formed four equally sized groups of images ranging from CD8^+^ T cell absence to high infiltration (Fig. 2A), and then observed the relationship between the composition of cells expressing functional factors and CD8^+^ T cell density (Fig. 2B). The proportions of cells expressing inflammatory cytokines (IL-1b and S100A9) and chemokines (CCR7, CXCL13) tended to be gradually increased with CD8^+^ T cell density. In addition, proportions of cells expressing pSS markers (such as, SSA, SSB, LEF1 and MMP9) were also gradually increased with CD8^+^ T cell density, in exception to IFN-γ (Fig. 2B). These results suggested that with the increase of pSS disease activity and severity, the infiltration abundance of CD8^+^ T cells gradually increased and was accompanied by the activation of inflammatory response.

### Overall immune cell composition of pSS patients varies from histopathology and SSA

**Fig. 3:**
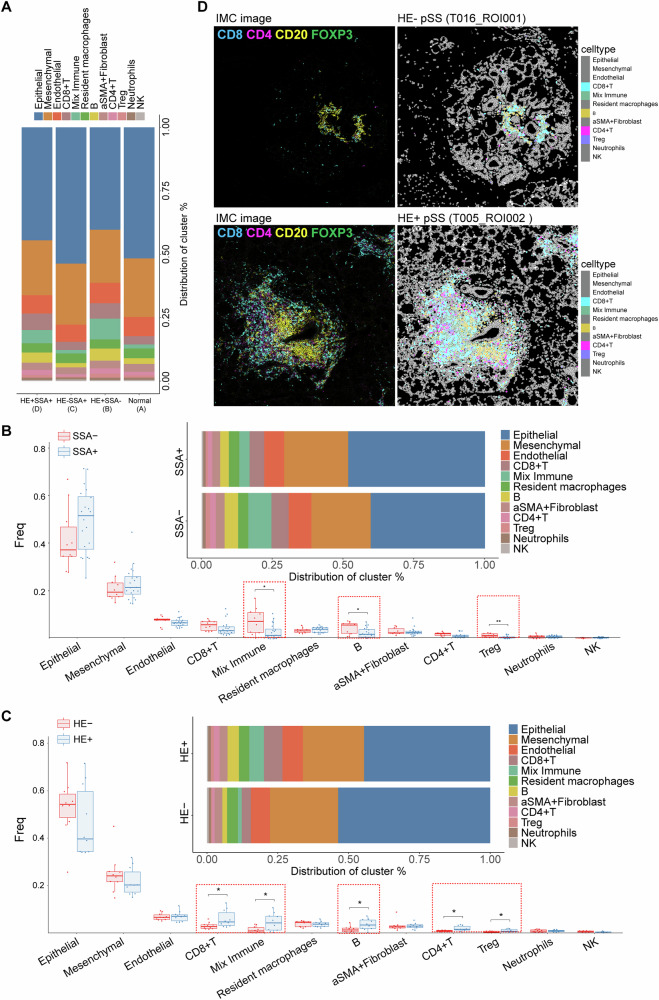
Cell type content analysis in different pSS subgroups. **A** Histogram demonstrating the differences on cell proportion in normal control and three pSS subgroups, including HE^+^SSA^–^, HE^–^SSA^+^ and HE^+^SSA^+^ pSS patients; **B** boxplots and histogram showing the differences on cell frequency and proportion of each cell type between SSA^+^ and SSA^–^ patients; **C** boxplots and histogram showing the differences on cell frequency and proportion of each cell type between HE^+^ and HE^–^ patients, *P < 0.05; **D** the IMC image and corresponding cell Voronoi map showing the distribution of differential immune cells types in representative HE^+^ and HE^–^ patients.

To characterize cellular changes during the development and progression of pSS, we assigned pSS patients into three groups, including HE^+^SSA^–^, HE^–^SSA^+^ and HE^+^SSA^+^ patients. As shown in Fig. 3A, the overall cell composition of HE^–^SSA^+^ patients (group C) were similarly to that of healthy controls (group A), while HE^+^SSA^–^ (group B) and HE^+^SSA^+^ (group D) patients showed similar overall cell composition. Particularly, HE^+^SSA^–^ (group B, 27.58%) and HE^+^SSA^+^ (group D, 23.77%) harbored high proportion of immune cells than HE^–^SSA^+^ patients (group C, 12.78%) and healthy controls (group A, 14.51%) (Fig. S4). This suggested that positive histopathology staining exhibited more influences to the overall cell composition and immune infiltration patterns, while SSA exhibited disparate influences on immune infiltration patterns from the positive histopathology. To further confirm this speculation, we compared the cellular changes of pSS patients divided by SSA and histopathology. Compared to SSA^–^ pSS patients, those with SSA^+^ exhibited significant lower infiltrating levels of mixed immune cell, B cells and Tregs (Fig. 3B). Besides, SSA^–^ pSS patients tended to show a high infiltration level of CD8^+^ T cells than those with SSA^+^. Whereas, multiple immune cells, including CD8^+^ T cells, CD4^+^ T cells, B cells, Treg and mixed immune cells showed significant differences between HE^+^ pSS patients and HE^–^ pSS patients (Fig. 3C). Specifically, infiltrating levels of these immune cells were significant higher in HE^+^ pSS patients than that of HE^–^ pSS patients. To evaluate the spatial organization of immune infiltration in pSS, the coordinate information of single cells extracted from each LSG tissue was utilized to generate cell distribution maps. The cell distribution maps effectively reflected the immune infiltration architecture observed in the corresponding IMC images (Fig. 3D).

### pSS patients unresponsive to treatment harbored a higher lymphatic infiltration and lower epithelial in LSG lesions

**Fig. 4:**
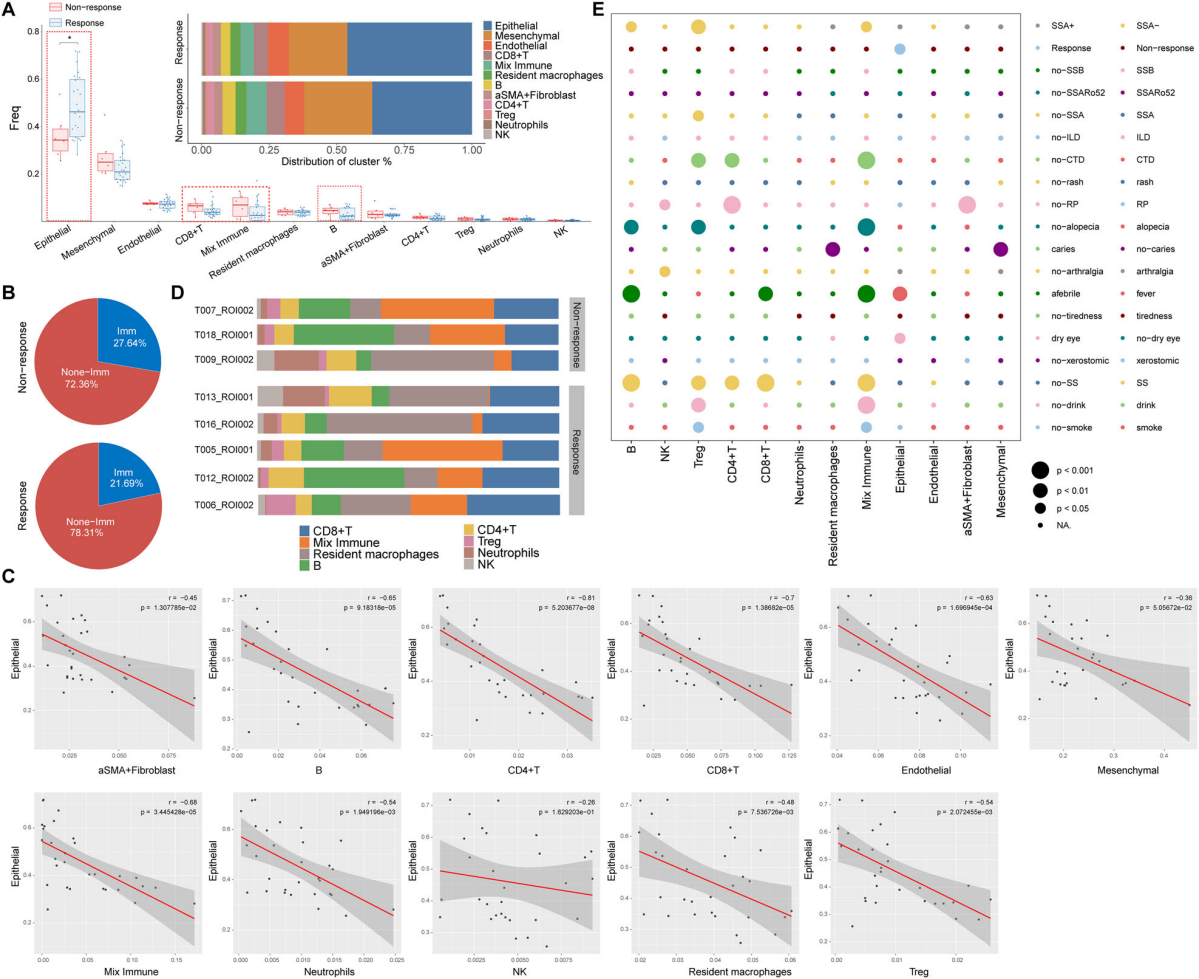
Correlations between immune cell composition and clinical features. **A** boxplots and histogram showing the differences on cell frequency and proportion of each cell type between response and non-response patients, *P < 0.05; **B** pie chart showing the percentage of immune and non-immune cells in response and non-response pSS patients; **C** Pearson’s correlations between the proportions of epithelial cells and other cells types across 30 ROIs of the 15 pSS patients. Each dot in the graphs represent one ROI; **D** histogram demonstrating the immune infiltration heterogeneity within lesions of response (five representative samples) and non-response; **E** bubble diagram showing the differences on the cell type content between subgroups divided by different clinical factors. This bubble plot shows the relationship between cell populations and clinical or pathological variables by interrogating the frequency of individual cell types as a percentage of total cells within each image. Circle size represents the level of significance. CTD connective tissue diseases; ILD interstitial lung disease; RP Raynaud’s phenomenon.

The 15 pSS patients were given conventional treatment with hydroxychloroquine sulfate tablets. After 6 months treatments, 12 patients were responsive to treatment with their ESSDAI reduced less than 4, and three patients were unresponsive to treatment. We further compared the cell composition among pSS patients responsive to treatment or not. Compared to response group, pSS patients in non-response group tended to have a higher proportion of CD8^+^ T, mixed immune and B cells (Fig. 4A), indicating a higher immune infiltration in their lesions. This was confirmed by the results in Fig. 4B. There was 27.64% overall immune cells proportion in non-response group, which was higher than the 21.69% in response group. Whereas, pSS patients in non-response group harbored a lower proportion of structural cells (72.36% vs. 78.31%) in their lesions when compared to those in response group. Such decrease on structural cells was mainly reflected in epithelial (Fig. 4A, B). Correlation analysis indicated that the proportion of epithelial cells in each ROI was negatively correlated with the proportion of all of the other cell types in exception of NK cells and mesenchymal (Fig. 4C). This suggested that the immune activation in LSG may cause the apoptosis of epithelial.

### Immune infiltration heterogeneity within LSG lesions and the correlations with clinical features

The three patients in non-response and five patients from the response group were selected to exhibit the high heterogeneity of immune cells composition. We observed a high degree of variability in terms of the immune cell composition within individual pSS patient LSG lesions (Fig. 4D). While all LSG lesions of pSS patients consistently had immune infiltrate, the infiltrating levels varied greatly (Fig. 4D). For example, among the three patients in non-response group, one patient (T009, HE^–^) had lower mixed immune and B cells and had a higher level of resident macrophages, neutrophils and NK cells, when compared to the other two patients (T007 and T018, HE^+^). This pSS patient (T009) combined with glomerular minor changes with globular sclerosis (Table S1). Interestingly, we found that two patients (T013 and T016) in response group exhibited similar immune cells composition to the T009 patient in non-response group (Fig. 4D), and these two patients were both HE^–^SSA^+^ pSS patients. To further illustrate the heterogeneity among pSS patients, their associations with clinical factors were investigated (Fig. 4E). Several immune cells, including B, CD4^+^ T, CD8^+^ T, Treg and mixed immune cells exhibited strong linkage with clinical factors, such as SS, fever, alopecia and connective tissue diseases (CTD). For example, CD4^+^ T, Treg and mixed immune cells varied greatly between pSS patients divided by CTD. Treg and mixed immune cells varied greatly between pSS patients divided by drink and smoke.

### Major cellular neighborhood changes in LSG microenvironment of pSS

**Fig. 5:**
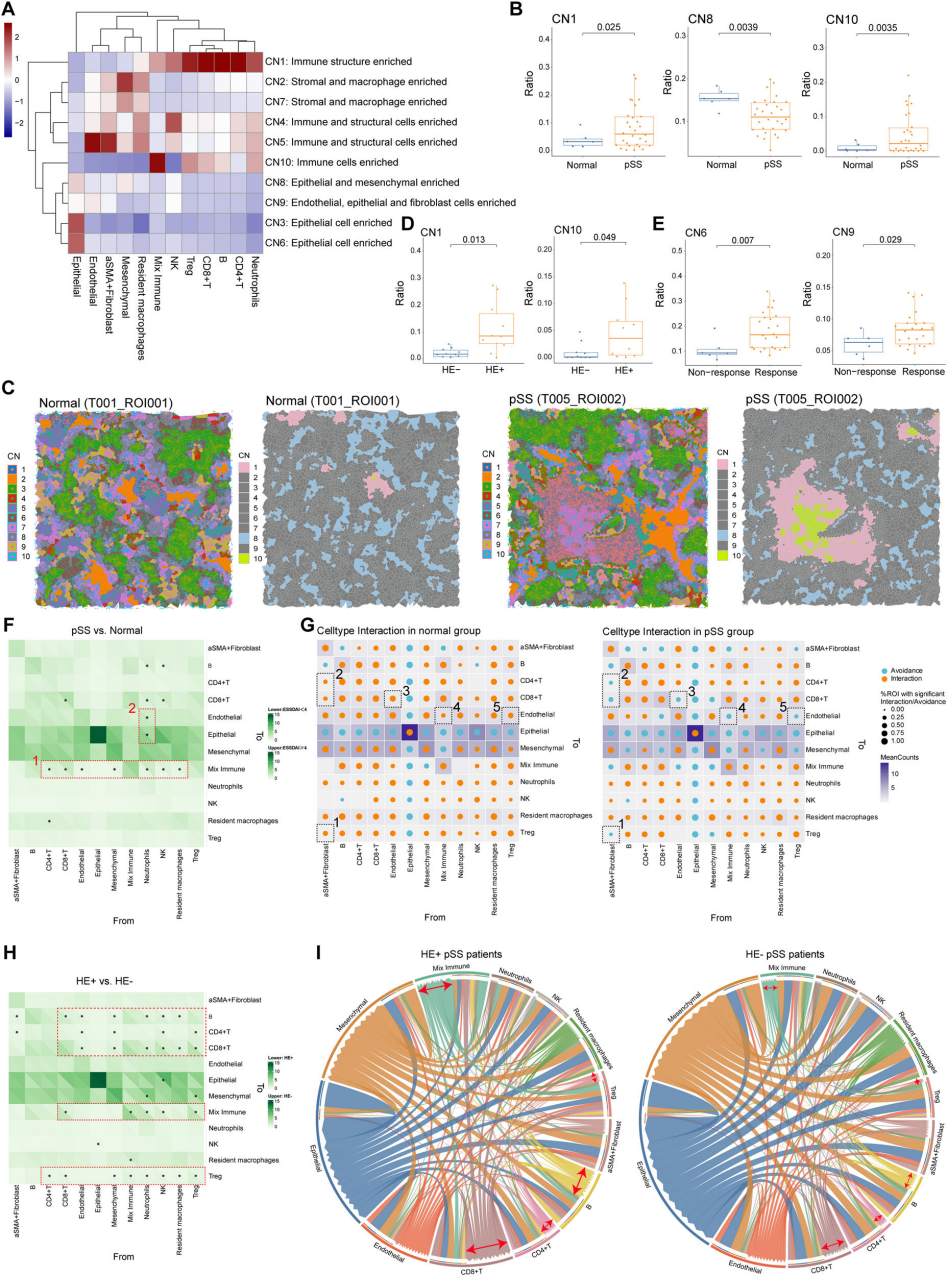
Changes in cellular neighborhood and cell–cell spatial interactions in pSS microenvironment. **A** Heatmap showing the ten distinct cellular neighborhoods (CN) identified based on the original cell types (the 12 identified cells in cell clustering) and their respective abundances within each CN. CN was defined by its center cell and the 20 nearest neighbor cells; **B** three CNs (CN1, CN8 and CN10) showed significant differences on their abundance between normal and pSS patients; **C** Representative Voronoi diagrams of CNs of normal and pSS patients. The distribution of all identified CNs (left) and the dysregulated CNs (right) were mapped onto the corresponding IMC images; **D** the abundance of the two dysregulated CNs (CN1 and CN10) between HE^+^ and HE^–^ pSS patients; **E** the abundance of the two dysregulated CNs (CN6 and CN9) between response and non-response pSS patients; **F** heatmap showing the differences on cell-cell communications counts between pSS and normal controls. The cell-cell spatial communications (interactions/avoidances) counting was defined as the number of each cell type within a CN of a certain cell, and the differences on communications between each cell type within each CN were compared by permutation test, *P < 0.05; **G** patterns of cell-cell interactions/avoidance for cell type interaction in normal and pSS groups. Orange and blue circles representing interactions pattern and avoidance pattern, respectively. The dotted boxes depict associations referenced in the text; **H** heatmap showing the differences on cell-cell communications counts between HE^+^ and HE^–^ pSS patients, *P < 0.05 in permutation test; **I** circle diagrams showing the specific cell-cell communications among different cell types. The red arrows depict the cell types with obvious differences between groups, as referenced in the text.

Elucidating the cell types and the cross-talk among these cells in pSS microenvironment may provide insights to decode the occurrence, progression and therapeutic response of pSS. Thus, we conducted the regional CN analysis as the methods described above. CNs were annotated based on the main cellular clusters to define the diverse the CN function units, and 10 CN function units were revealed (Fig. 5A). In the LSG microenvironment of pSS patients, the ratio of CN1 (immune structure enriched) and CN10 (immune cells enriched) were markedly enhanced, while the ratio of CN8 (epithelial and mesenchymal enriched) was markedly decreased when compared to those of normal controls (Fig. 5B). A Voronoi plot was employed to visualize the CN components in the LSG microenvironment of both normal control and pSS patients. Consistently with the findings in Fig. 5B, we observed more CN1 and CN10 components in the LSG microenvironment in the pSS patient (Fig. 5C). This indicated an enhanced immune infiltration in LSG lesions of pSS. We further characterize the CN composition in pSS patients. Between SSA^+^ and SSA^–^ pSS patients, no obvious changes on composition of CN1-9 were observed, whereas there was a higher ratio of CN10 (immune cells enriched) in SSA^–^ pSS patients than that of SSA^+^ patients (Fig. S5). This was also supported by the findings obtained from immune cells composition between SSA^+^ and SSA^–^ pSS patients in Fig. 3B. While, significantly enhanced ratio of CN1 and CN10 were observed in HE^+^ pSS patients than that in HE^−^ pSS patients, as expected (Fig. 5D). Notably, pSS patients in response group exhibited high ratio of CN6 (epithelial cell enriched) and CN9 (Endothelial, epithelial and fibroblast cells enriched) than those in non-response group (Fig. 5E), which was agreed with the findings that pSS patients in response group harbored a higher proportion of structural cells in their lesions.

### Spatial interaction pattern among cells contributed to the heterogeneity of the pSS microenvironment

To further illustrate the specific cellular communications within CNs in pSS microenvironment, the interactions among diverse types of cells were investigated. Overall, the pSS lesions showed more interactions among immune cells than that of normal controls (Fig. 5F). For example, there were more interactions from immune cells (such as CD4^+^ T, CD8^+^ T, neutrophils and NK cells) to mixed immune cells (dotted box 1), suggesting an immunoactive state. While there were more interactions between neutrophils-epithelial and neutrophils-endothelial in LSG of normal controls (dotted box 2, Fig. 5F). Permutation test revealed the changes on spatial interaction pattern among cells (Fig. 5G). Specifically, the interaction pair between α-SMA^+^ fibroblast to several immune cells (Treg, CD8^+^ T and CD4^+^ T cells, dotted box 1–2), the interaction pairs between endothelial and CD8^+^ T cell (dotted box 3), mix immune and endothelial (dotted box 4), and Treg and endothelial (dotted box 5), changed from the interaction pattern in normal control group to avoidance pattern in the pSS group (Fig. 5G). The cellular communications pattern was also explored in different pSS patients. The interactions of mix immune with resident macrophages and NK cells were markedly differed between SSA^+^ and SSA^–^ patients. Concretely, the interactions from mix immune to resident macrophages and NK cells in SSA^+^ patients were changed to avoidance pattern in SSA^+^ patients (Fig. S6A, B). Besides, compared to SSA^–^ patients, the number of interactions from several cells (such as epithelial cells, stromal cells, CD4^+^ and CD8^+^ T cells) to Treg cells was significantly reduced in SSA^+^ patients (Fig. S6A, B). This might be attributed to the reduced infiltration levels of Treg cells in SSA^+^ patients, as shown in Fig. 3B. Between HE^+^ and HE^–^ pSS patients, there were large scale of changes on cellular communications (Fig. 5H). Specifically, there were more cellular interactions from various immune cells to mixed immune, CD8^+^ T, CD4^+^ T, B and Treg cells in HE^+^ pSS patients (Fig. 5H, I). Besides, significant differences were detected between response and non-response groups in terms of the cellular interactions across several cells to epithelial, but no between other cells (Fig. S6C). Notably, the interaction of neutrophil to several cells (CD4^+^ T cells, endothelial and mesenchymal), changed from the interaction pattern in response group to avoidance pattern in non-response group (Fig. S6D). These findings contributed to illustrate the heterogeneity of the pSS microenvironment.

### CD8^+^ T cells dominated the most of the changed intercellular interactions in pSS

**Fig. 6:**
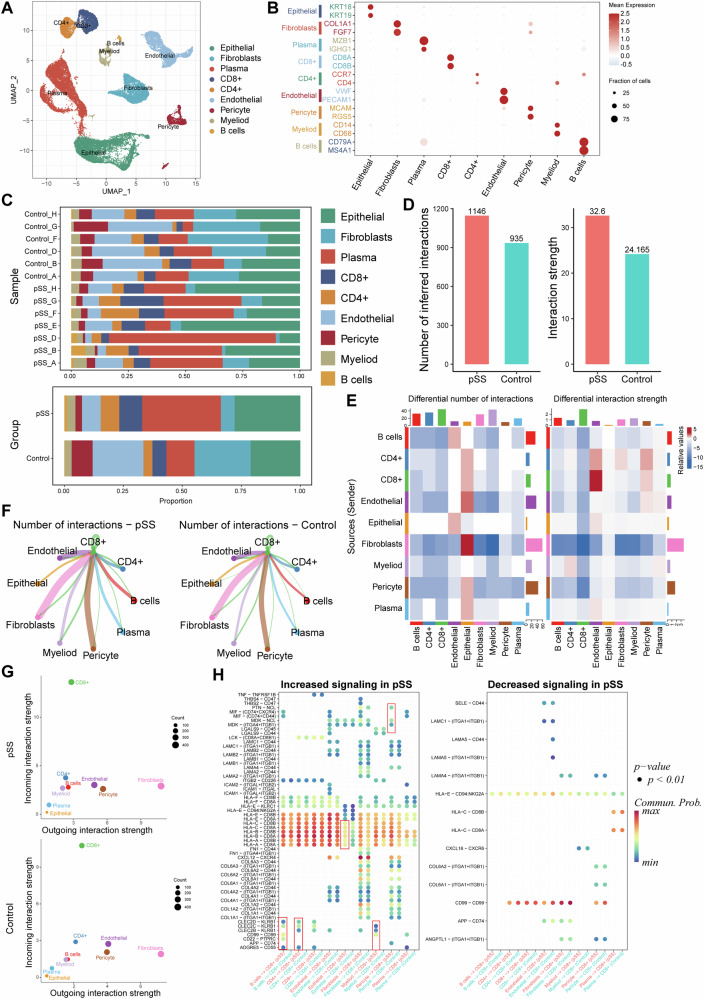
Intercellular ligand–receptor interactions in pSS. **A** The UMAP cell clustering based on the GSE272409 dataset, in which the captured cells were clustered into 9 cell populations; **B** expression of specific cell markers in each cell type; **C** Histogram demonstrating the differences on cell proportion in each normal control and pSS patient (upper, shown by samples) as well as in two groups (bottom, shown by groups); (**D**), **E** The total interactions numbers and strength in pSS and control samples; **E** the intercellular interactions numbers and strength in pSS and control samples; **F** interaction numbers of CD8^+^ T cells with other cell types, with a thick line representing more interaction numbers; **G** bubble diagram showing of incoming and outgoing signaling strength of each cell type for pSS (upper) and control samples (bottom). The Y-axis and X-axis presents the incoming and outgoing interaction strength, respectively; **H** bubble diagrams showing the increased and decreased interactions from receptors of other cell types to the ligands of CD8^+^ T cell. The red boxes depict associations referenced in the text.

Based on the sc-RNA seq data, we identified and annotated nine cells clusters, including B, CD4^+^ T, CD8^+^ T, endothelial, epithelial, fibroblast, myeloid, pericyte and plasma cells (Fig. 6A, B). The infiltrated immune cells were dominated mainly by CD4^+^ and CD8^+^ T lymphocytes as well as B cells, with their proportion increased in pSS samples than in controls (Fig. 6C). Besides, the proportion of endothelial, fibroblast and pericyte were decreased, whereas the proportion of epithelial and plasma cells were increased in pSS samples compared to that in controls (Fig. 6C). Notably, there was significant heterogeneity in cell proportions among pSS samples (Fig. 6C). The intercellular communications were further analyzed. In comparison with control samples, there were more intercellular ligand–receptor interactions and an enhanced interaction strength in LSG of pSS patients (Fig. 6D). Such differences in both interaction numbers and strength between pSS and controls were mainly reflected in CD8^+^ T cells (Fig. 6E). Hence, we further investigated the interactions from CD8^+^ T cells to other cells. Endothelial, fibroblast and pericyte were identified as the cells that frequently interacted with CD8^+^ T cells in both pSS and controls (Fig. 6F). Among these intercellular ligand-receptor interactions, the most of the incoming interactions were dominated by CD8^+^ T cells (Fig. 6G), indicating there were more intercellular communications from other cells to CD8^+^ T cells through the ligand-receptor interactions. The significantly changed ligand signaling from other cells to the receptors of CD8^+^ T cells were further revealed (Fig. 6H). Majority of ligand–receptor interactions from other cells to CD8^+^ T cells were increased in pSS patients. For example, the CLEC2C- KRB1 ligand–receptor interactions from B cells and CD4^+^ T cells to CD8^+^ T cells were present in pSS patients but not in controls. Particularly, the HLAs-CD8A/B ligand–receptor interactions from other cells to CD8^+^ T cells were overall increased in pSS patients.

### Phenotypic transition of CD8^+^ T cells in the progression of pSS

**Fig. 7:**
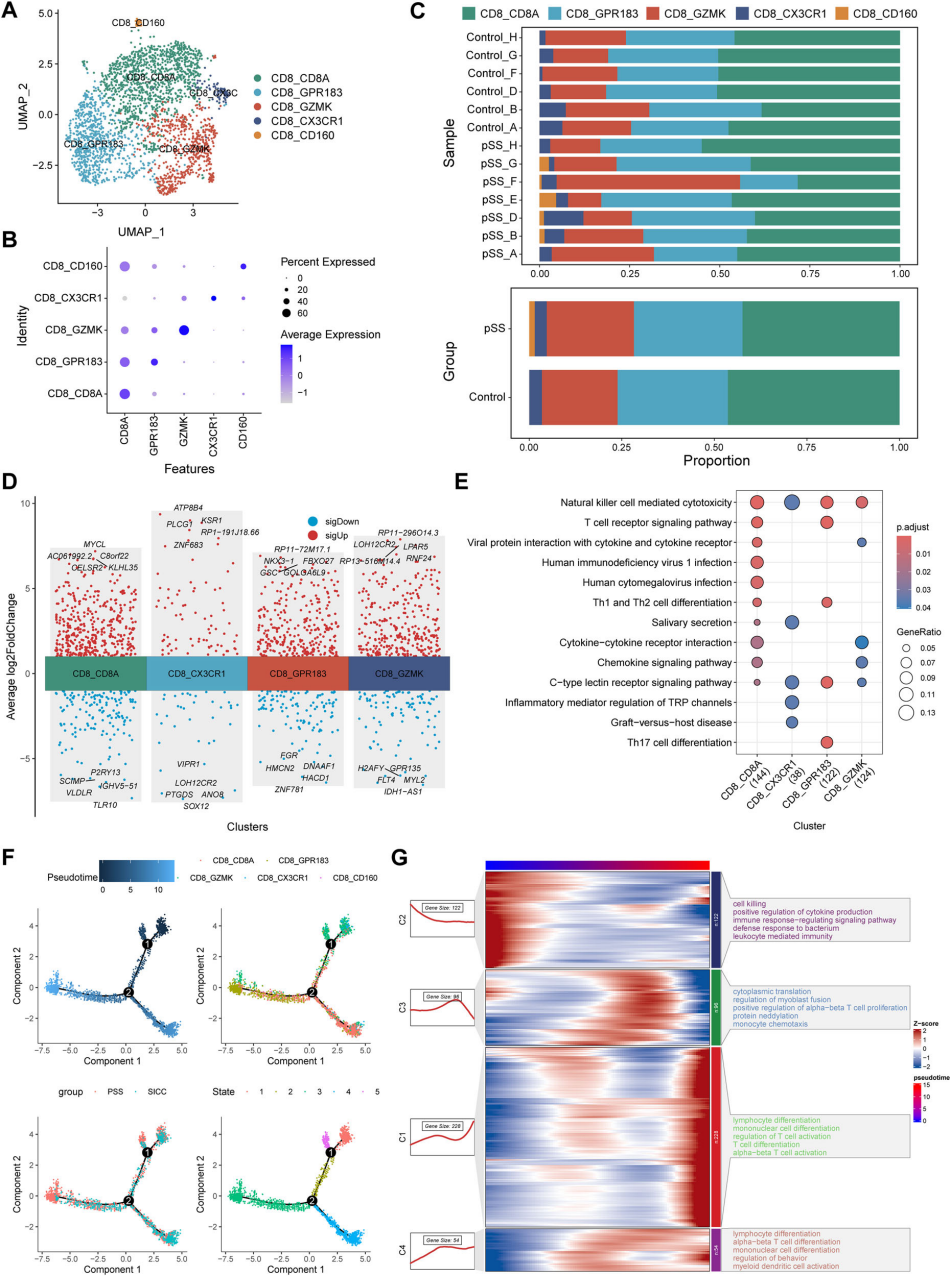
CD8^+^ T cell subset identification and pseudotime trajectory analysis. **A** The UMAP cell clustering of CD8^+^ T cells in GSE272409 dataset, in which the CD8^+^ T cells were clustered into five cell subsets; **B** expression of cell markers in each CD8^+^ T cell subset; **C** Histogram demonstrating the differences on cell proportion in each normal control and pSS patient (upper, shown by samples) as well as in two groups (bottom, shown by groups); **D** the top 5 upregulated and downregulated genes in each CD8^+^ T cell subset (in exception to CD160^+^CD8^+^ T cells because that this cell subset appeared to present only in pSS patients); **E** functional enrichment of the genes in each CD8^+^ T cell subset; **F** pseudotime trajectory analysis of CD8^+^ T cell subsets, and the results were shown as per Pseudotime, cell clusters, groups and state in turn; **G** heatmaps showing the changes on functions of CD8^+^ T cell subsets along with the pseudotime trajectory.

Totally, 2559 CD8^+^ T cells were extracted from the dataset, and five subsets of CD8^+^ T cells were identified after cell clustering and annotation [37], including CD8A^+^CD8^+^ T cells, GPR183^+^CD8^+^ T cells (central memory T cells, T_CM_), GZMK^+^CD8^+^ T cells (effector memory T cells, T_EM_), CX3CR1^+^CD8^+^ T cells (recently activated effector memory or effector T cells, T_EMRA_/T_EFF_) and CD160^+^CD8^+^ T cells (intraepithelial lymphocytes, IEL) (Fig. 7A, B). CD160^+^CD8^+^ T cells subset appeared to present only in pSS patients, and the proportion of GZMK^+^CD8^+^ T cells was increased in pSS patients (Fig. 7C). Differential expression genes between pSS and control was conducted for each cell subset (Fig. 7D) in exception to CD160^+^CD8^+^ T cells, followed by functional enrichment. The results suggested that all the four cell subsets were implicated in NK cell mediated cytotoxicity. Besides, GPR183^+^CD8^+^ T cells cells were mainly involved in T cell receptor signaling pathway, Th1, Th2 and Th17 cell differentiation (Fig. 7E). To further investigate the roles of different CD8 + T cells subsets in the progression of pSS, we performed trajectory analysis (Fig. 7F). Five differentiated states were observed, and the GZMK^+^CD8^+^ T cells were mainly concentrated in State 1, while GPR183^+^CD8^+^ T cells were mainly concentrated in State 3. Meanwhile, there were more cells in State 3 in pSS, implying that CD8^+^ T cells in the progression of pSS were mainly in a central memory phenotype. During the differentiation processing of CD8^+^ T cells, the dysregulated genes were screened, and these genes were clustered into four groups based on their expression pattern (Fig. 7G). Genes highly expressed in the initial differentiation state were implicated in cytokine production, leukocyte mediated immunity and immune response−regulating signaling pathway. Whereas genes highly expressed in the terminal differentiation state were mainly involved in T cell activation and lymphocyte differentiation processes (Fig. 7G). These findings suggested that CD8^+^ T cell differentiation and activation were more frequent during the progression of pSS.

### CD160 expression on CD8^+^ T cells was associated with the injury of SGECs

**Fig. 8:**
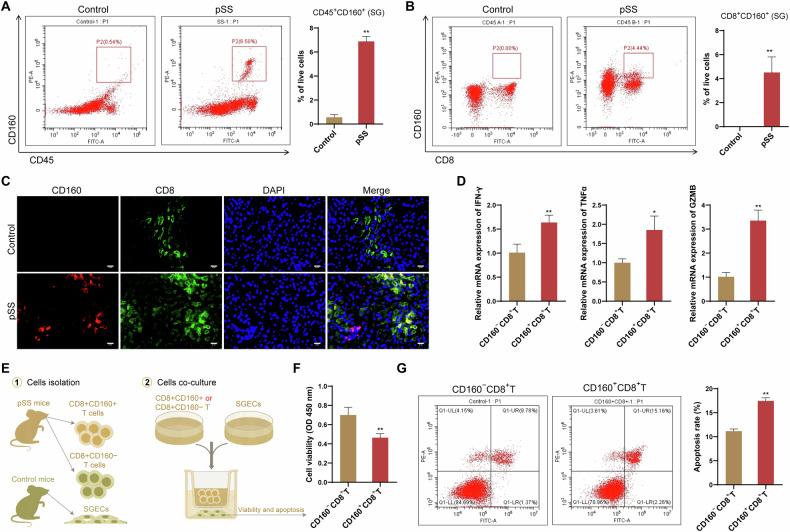
CD8^+^CD160^+^ T cells contributed injury of SGECs. **A** Representative data showing CD45^+^CD160^+^ cells in the SG of NOD/Ltj mice (pSS model) and control mice by flow cytometry; **B** Representative data showing CD8^+^CD160^+^ cells in the SG of NOD/Ltj mice (pSS model) and control mice by flow cytometry; **C** Representative immunofluorescence images of CD8 (green) and CD160 (red) staining in the SG of NOD/Ltj mice (pSS model) and control mice; **D** The mRNA expression of IFN-γ, GZMB and TNF-α in CD8^+^CD160^+^ T cells relative to CD8^+^CD160^−^ T cells; **E** Experimental scheme for the co-culture of stimulated CD8^+^CD160^+^ T or CD8^+^CD160^−^ T cells with the SGECs; **F** cell viability of SGECs determined by CCK-8 assay; **G** apoptosis of SGECs determined by flow cytometry. *P < 0.05, **P < 0.01.

The potential role of CD160^+^CD8^+^ T cells in pSS pathogenesis was further investigated. CD160 expression on flow-sorted CD45^+^ cells from the SG of NOD mice were first examined. Compared with control mice, NOD/Ltj mice showed a significant high expression of CD160 on CD45^+^ cells (Fig. 8A). Among these CD45^+^ cells, further investigation indicated that the enhanced CD160 expression in SG of NOD/Ltj mice was mainly presented on CD8^+^ T cells (Fig. 8B). Specifically, NOD/Ltj mice showed an obvious colocalization of CD160 and CD8 in their SG tissues, while such colocalization was not detected in SG tissue of control mice (Fig. 8C). This was consistent with the findings from sc-RNA seq analysis, that is, CD160^+^CD8^+^ T cells subset appeared to present only in pSS patients. Additionally, expression of IFN-γ, GZMB and TNF-α was found to be enhanced in CD8^+^CD160^+^ T cells relative to CD8^+^CD160^−^ T cells (Fig. 8D), indicating a potential proinflammatory and cytotoxic role of SG CD8^+^CD160^+^ T cells in the pathogenesis of pSS. This was also evidenced by the results of coculturing of the CD8^+^CD160^+^ T cells with SGECs. As shown in Fig. 8E, F, SGECs that cocultured with the CD8^+^CD160^+^ T cells showed a reduced cell viability and an enhanced ratio of apoptosis.

## Discussion

As one of the most common autoimmune diseases, pSS harbors complex and unclear pathogenesis yet [38]. Salivary gland is not only the organ affected in pSS disease, but also an important place to drive the development of pSS [39, 40]. Therefore, in-depth exploration of the pSS salivary gland microenvironment components and the cellular interactions will contribute to better understand the pathogenesis of pSS and provide more valuable targets for drug development. Although all salivary glands are affected in pSS, LSG is commonly used in diagnosis and research because it is relatively easy to obtain [40]. Hence, based on the IMC analysis of LSG samples, we provided a comprehensive characterization of cell constitutions and intercellular communications in pSS lesions in the current study.

As a systemic autoimmune disease, pSS exhibit histologic hallmark of lymphocyte infiltration. Particularly, T cells are regarded as the primary contributor to the onset and progression of pSS [41, 42]. Nevertheless, the immunological and pathogenic properties of pSS remain unclear largely. Reportedly, at the onset of pSS, autoantigens (such as Ro/SSA and La/SSB) derived from the salivary glands may induce a chronic inflammatory microenvironment that drives the activation of T cells [39]. Activated T cells, particularly CD4^+^ T cell subsets, such as follicular helper T (Tfh) and Tfh-like cells, contribute to pathogenesis by generating pro-inflammatory cytokines and by triggering B cell activation [42, 43], which leads to the establishment of a positive feedback loop [41]. Our IMC analysis demonstrated that CD8^+^ T cells, but not CD4^+^ T cells, were the most prominent T cells in immune infiltrates of pSS LSG. A previous study also reported an enhanced infiltrating number of activated CD8^+^ T cells in salivary gland of pSS patients by CyTOF analysis [24]. Similarly, based on sc-RNA seq analysis, Xu et al. also indicated that CD8^+^ T cells underwent significant clonal expansion in pSS patients, and mainly proposed the pathogenic role of GZMK^+^CD8^+^ T cells in pSS [44]. However, the role of CD8^+^ T cells in pSS has rarely been explored. Emerging evidences demonstrate that CD8^+^ T lymphocytes exert important roles in acinar injury in the exocrine glands [45–47]. Several bioinformatic investigations suggest that CD8^+^ T cells are associated with the pathology and severity of pSS [44, 48]. In pSS mice model, depletion of CD8^+^ T cells was found to markedly attenuate the tissue damage and improve the salivary gland functions [49]. We further observed the relationship between CD8^+^ T cell density and the composition of cells expressing functional factors. The results showed that proportions of cells expressing inflammatory/chemokines cytokines and several pSS markers such as MMP9 tended to be gradually increased with CD8^+^ T cell density. Extracellular matrix (ECM) represents the major structure fundamental to epithelial homeostasis and the maintaining of salivary gland epithelial cell function, and its degradation is a trait early in pSS pathophysiology [50]. MMP9, a key matrix metalloproteinase, plays important roles in perpetual degradation and remodeling of ECM in pSS, and the glandular expression and activity of MMP9 is strongly linked to the degree and severity of salivary gland damage and functional changes in pSS [51, 52]. The above findings suggested that with the increase of pSS disease activity and severity, the infiltration abundance of CD8^+^ T cells gradually increased and was accompanied by the activation of inflammatory response. Therefore, targeting CD8^+^ T cells might be a rational strategy for the treatment of pSS.

We further characterized CD8^+^ T cells based on scRNA-seq data for pSS. CD8^+^ T cells dominated the most intercellular ligand-receptor interactions, and the differences in both interaction numbers and strength between pSS and controls were mainly reflected in CD8^+^ T cells. Particularly, endothelial, fibroblast and pericyte were identified as the cells that frequently interacted with CD8^+^ T cells. Five CD8^+^ T cells subsets were annotated. CD160^+^CD8^+^ T cells subset appeared to present only in pSS patients, and proportion of GZMK^+^CD8^+^ T cells was increased in pSS patients. A previous study demonstrated that the enriched GZMK^+^CD8^+^ T cells in human inflamed tissue were the major inflammatory cytokine producer, which exerted crucial roles to drive inflammation [53]. Therefore, it is reasonable to speculate that the increased proportion of GZMK^+^ CD8^+^ T cell subsets might be responsible for the increase in inflammatory cytokine levels in pSS patients. CD160 is an Ig-like glycoprotein frequently expressed on circulating NK cells, γδ T cells and other immune cells. As for CD8^+^ T cells, CD160 is reported to express frequently on the CD8^+^ memory T cells and recently activated CD8^+^ T cells, and CD160^+^CD8^+^ T cells produce IFN-γ more rapidly compared to that of CD160^–^CD8^+^ T cells under antigen stimulation [54]. CD160 may exert its actions on CD8^+^ T cells in several ways, including facilitating proliferative capacity, promoting the escape of CD8^+^ T cells from terminal differentiation and enhancing the cytotoxicity of CD8^+^ T cells [55]. Consistently with these reports, our in vitro experiments indicated that CD8^+^CD160^+^ T cells showed an enhanced expression of IFN-γ, GZMB and TNF-α relative to CD8^+^CD160^−^ T cells upon stimulation. An increased GZMK expression on CD8^+^ T cell subset was also observed in study of Huang et al. [56], indicating an enhanced cytotoxic activity of this subset in pSS. Besides, CD8^+^CD160^+^ T cells could hamper the viability while promote the apoptosis of SGECs. These findings suggested that CD8^+^CD160^+^ T cells exerted a potential proinflammatory and cytotoxic role of SG CD8^+^CD160^+^ T cells in the pathogenesis of pSS. Targeting CD8^+^ T cells, such as depletion of CD8^+^ T or inhibition of activity of CD8^+^ T cells, may be a rational therapeutic strategy for human pSS.

Besides, we also observed an enhanced infiltration of B cells, Tregs and a mixed immune cells population. Overactivation of B cells is a characteristic manifestation of pSS [57], which produces a large number of autoantibodies, some of which can bind to autoantigens to generate immune complexes, which are deposited in multiple organ systems and cause damage to corresponding tissues [58]. Besides, in the process of pSS, B cells can secrete a variety of pro-inflammatory factors and present antigens to T cells. Part of the pSS patients in tertiary lymphatic gland tissue structure (tertitarylymphoidstructures, TLS) or ectopic germinal center (germinal center, GC) sample structure, promote autoimmunity, glandular tissue destruction [58]. The mixed immune cells could be explained as an immune complex structure, in which various immune cells closely interacted with each other, indicating a state of immune activation. Regarding the involvement of Treg cells in pSS, data from a variety of studies launched conflicting information, and the associations across infiltrating Treg cells and clinical features have largely unclear [41, 59]. Data from the current study revealed that levels of infiltrating Tregs varied between pSS patients divided by clinical factors, such as smoke, drink and CTD, implying the associations of infiltrating Tregs with clinical feature.

We observed that epithelial was the main cell type in LSG of pSS, which was also observed in the single-cell study by Xiang et al. [22]. SGECs are the main cells that produce and transport saliva in the salivary gland microenvironment, and are also the important target cells that drive the initiation of the autoimmune responses [60, 61]. Considering the widespread involvements of epithelial cells in pSS, the terms of autoimmune epithelitis is proposed [62, 63]. The inflammation is linked to the persistence of various inflammatory signals, such as interferon-gamma, whose elevated levels in pSS cause the death of SGECs [64, 65]. In the current study, we observed a decrease in epithelial proportion in LSG of pSS patients compared to the controls, which implied the presence of SGECs damage in pSS, conforming to the common clinical symptom of decreased salivary gland function in pSS. Interestingly, pSS patients who responsive to treatment harbored an obvious higher proportion of epithelial cells in their LSG than those unresponsive to treatment, highlighting the importance of epithelial cells in the treatment of pSS.

The communication between epithelial cells and immune cells is an important link in the pathogenesis of pSS [66, 67]. On the one hand, inflammatory factors derived from immune cells promote SGECs inflammatory response and tissue damage, and aggravate the impaired salivary secretion function. On the other hand, antigen presentation and immunoactive molecules expressed by SGECs promote immune cell activation and autoantibody formation [67]. Consistently, we observed that numbers of interactions from various cells (such as CD4^+^ T, Treg and resident macrophages) to epithelial varied greatly between response and non-response pSS patients. Therefore, illustration the communication between epithelial cells and immune cells might provide insights for developing treatment strategy in pSS. Besides, we also observed the stromal cells (mainly α-SMA^+^ fibroblast and endothelial) showed changed communication pattern with immune cells in LSG microenvironment of pSS and normal controls, manifested as the change from interaction pattern to avoidance pattern in pSS. These findings also implied the involvements of salivary glands stromal cells in the development and progression of pSS.

Limitations and prospects. First, we must admit that the findings from this study should be interpreted in the context of our IMC antibody panel. This also remains the inherent limitation of IMC studies. To more comprehensively characterize the immune microenvironment and underlying cellular communication in LSG of pSS, other cells types that may exert crucial roles in the onset and progression of pSS should be incorporated into the IMC antibody panel in future. Our study was also limited by clinical heterogeneity within patients (such as clinical manifestation and therapy status) and the small sample size. In future, we should prospectively collect a clinical cohort with large sample size and detailed pathological features, and focus mainly on the changes in each cell type at different stages of the disease to analyze their interactions and causal roles in disease onset and activity. Meanwhile, further analysis based on the comparisons between fresh tissue and the matched blood to identify the potential blood biomarkers for easy of clinical use.

## Conclusion

In summary, this study provided single cell profile with spatial information for analysis the LSG immune microenvironment in pSS, which could not be achieved by conventional immunofluorescence and immunohistochemistry assays. We identified 12 distinct cell populations involved in pSS and highlighted CD8^+^ T cells were the most prominent T cells in immune infiltrates of pSS LSG. Besides, Our IMC analysis also emphasized the importance of epithelial cells, and the communication between epithelial cells and immune cells in the treatment of pSS. This study provided insights for targeted therapies based on the molecular and single-cell characteristics of the major cell types in the diseased LSG.

## Supplementary information

Supplementary materials

Supplementary Table 1

Supplementary Table 2

Supplementary Table 3

## Data Availability

The imaging mass cytometry data and any additional information required to reanalyze the data reported in this paper will be made available to other researchers upon reasonable request to the corresponding author. The single-cell RNA seq data can be obtained from the Gene Expression Omnibus (https://www.ncbi.nlm.nih.gov/geo/, accession no. GSE272409).
