# Supplementary material for: Application of imaging mass cytometry for spatially profiling the microenvironment of salivary glands in primary Sjögren’s syndrome
Source: Cell Death Dis. 2025 May 16;16(1):392. doi: 10.1038/s41419-025-07717-7 (PMC12084299; doi:10.1038/s41419-025-07717-7)
Supplement: Supplementary file 1 — Supplementary materials [file 41419_2025_7717_MOESM1_ESM.docx]

**Application of imaging mass cytometry for spatially profiling the microenvironment of salivary glands in primary Sjögren's syndrome**

**Running title:** Spatial proteomic profiling of pSS microenvironment

Guolin Wu^1*#^, Fangping Wu^2*^, Lipei Wang^3^, Lixiong Ying^4^, Wenwen Lu^1^, Kang Qian^1^, Tianxiao Fu^1^, Danbin Wu^1^, Fenglin Hu^1^, YiHang Shi^1^, Li Xu^1^

^1^Department of Traditional Chinese Medicine, The First Affiliated Hospital, Zhejiang University School of Medicine, Hangzhou, Zhejiang, China

^2^School of Pharmaceutical Sciences, Zhejiang Chinese Medical University, Hangzhou, Zhejiang, China

^3^Hangzhou Normal University of Basic Medical Sciences, Hangzhou, Zhejiang, China

^4^The First Affiliated Hospital, Zhejiang University School of Medicine, Hangzhou, Zhejiang, China

*These authors contributed equally to this work.

#**Corresponding Author:** Guolin Wu

Address: Department of Traditional Chinese Medicine, The First Affiliated Hospital, Zhejiang University School of Medicine, Hangzhou, Zhejiang, China

Email: wuguolin28@zju.edu.cn

Tel: +8613136150848

**Supplementary materials include 3 tables and 6 figures.**

**Supplementary Tables**

**Table S1.** The detailed clinical information of each included subjuect.

**Table S2.** The antibody panel of IMC analysis and the source of the antibodies.

**Table S3.** The used primers in RT-qPCR.

**Supplementary Figures**

**
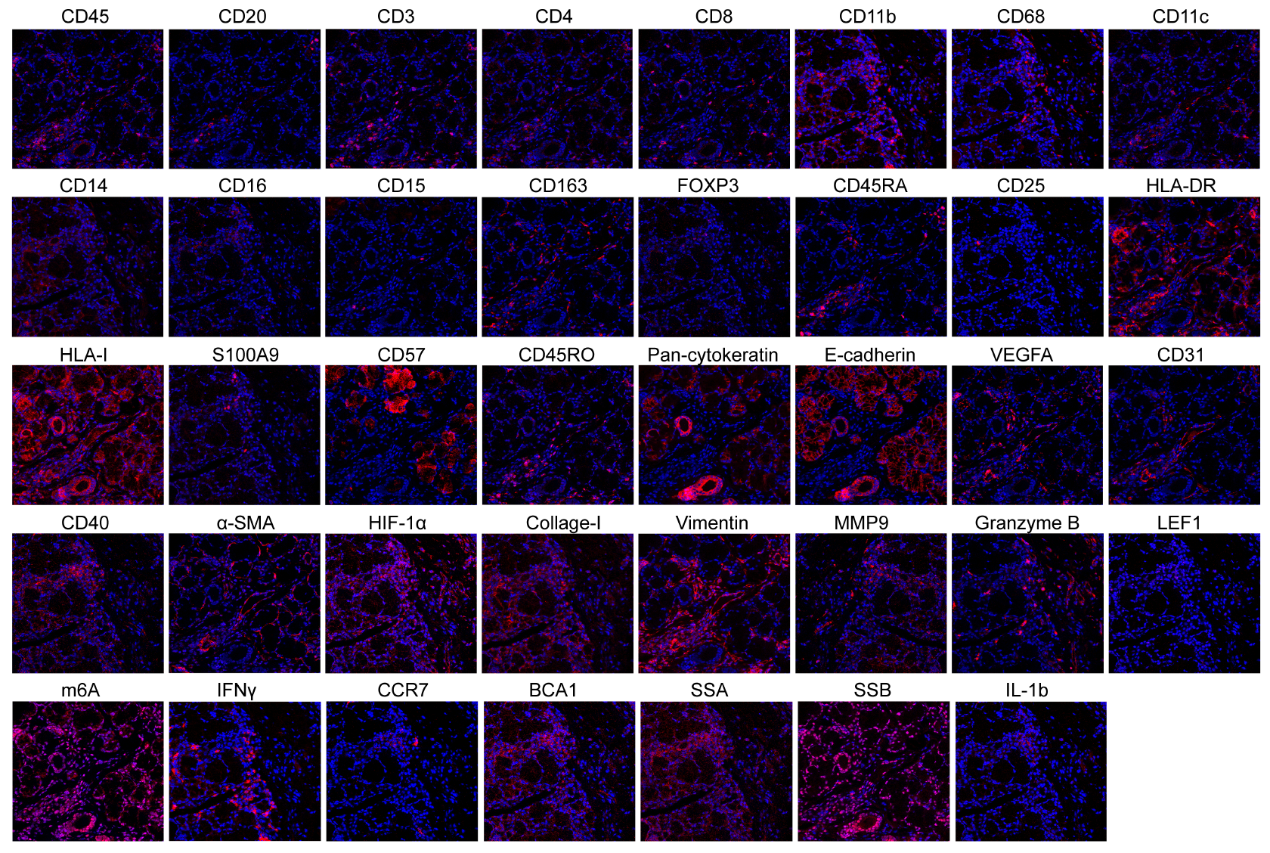
Fig. S1.** Single color staining of each marker in multiplexed antibody panel**.** Representative plot from 18 samples, with 2 independent experiments.

**
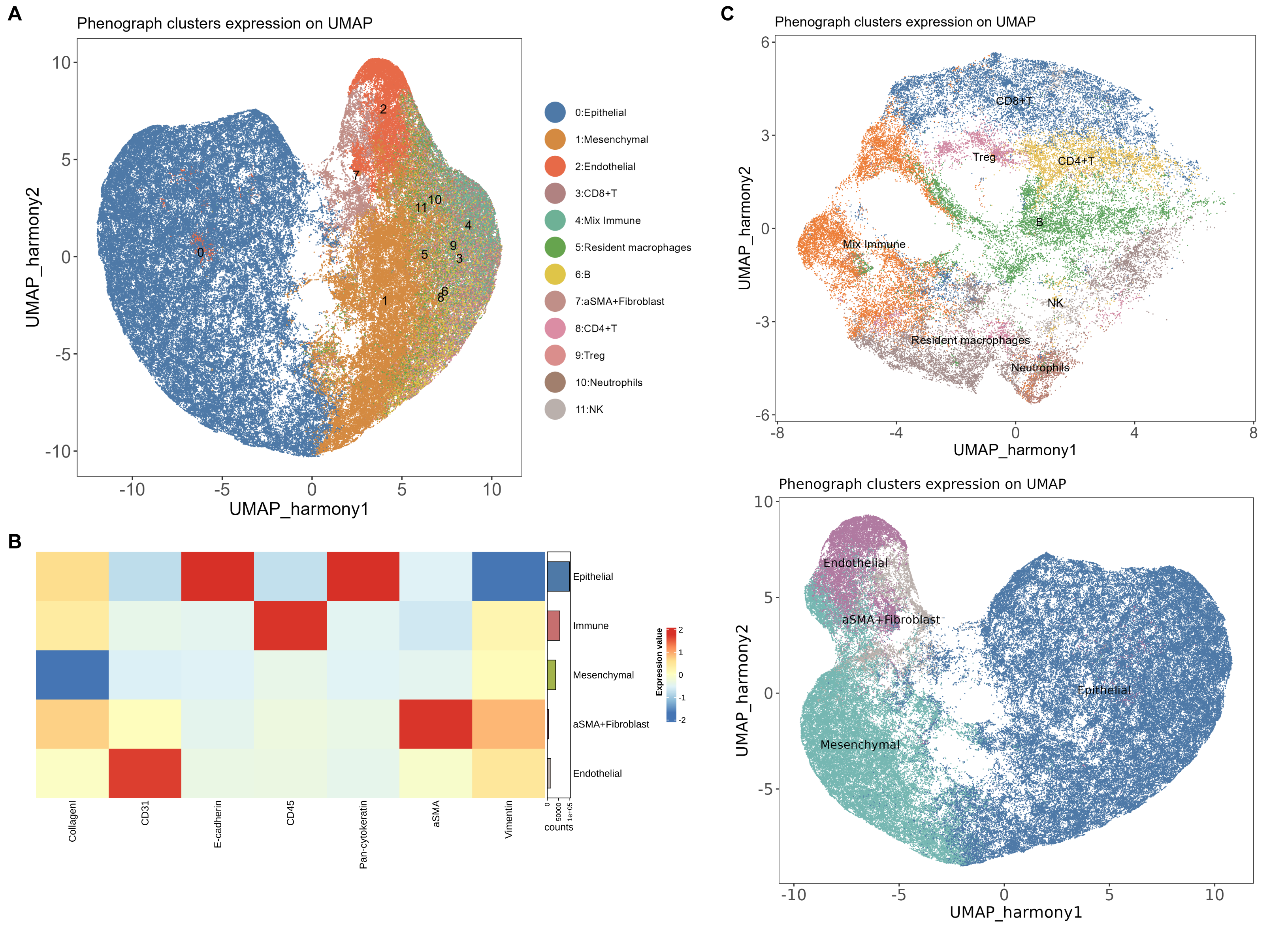
Fig. S2.** Dimensionality reduction clustering of cells. A, the UMAP of all captured cells, which were clustered into 12 cell populations; B, clustering heatmap showing the expression of major cell markers; C, the UMAP of eight types of immune cells (upper) and 4 types of non-immune cells (bottom).

**
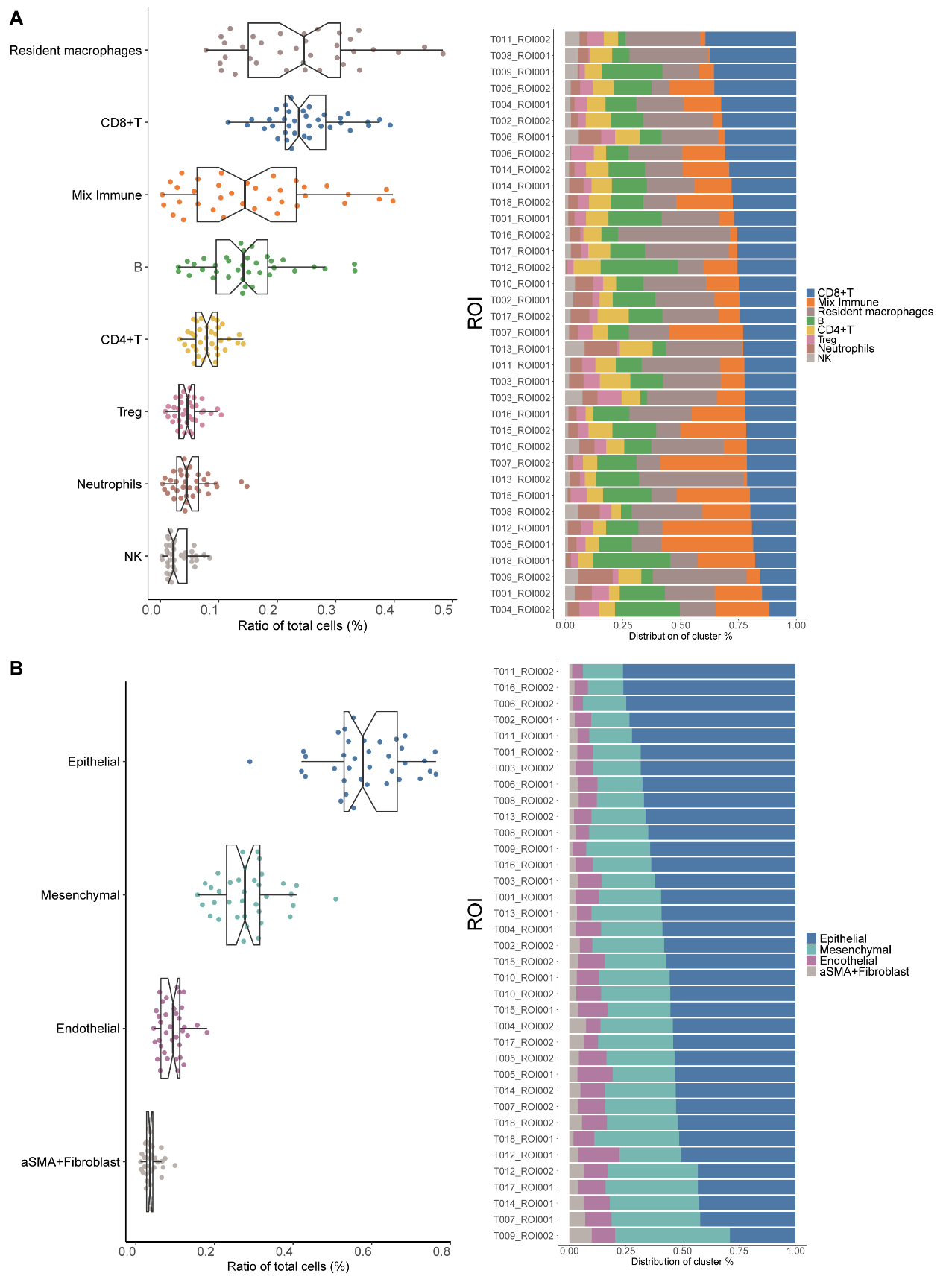
Fig. S3.** Cell type content analysis. A, the ratio and distribution of eight immune cell types in ROIs of all samples; B, the ratio and distribution of four non-immune cell types in ROIs of all samples

**
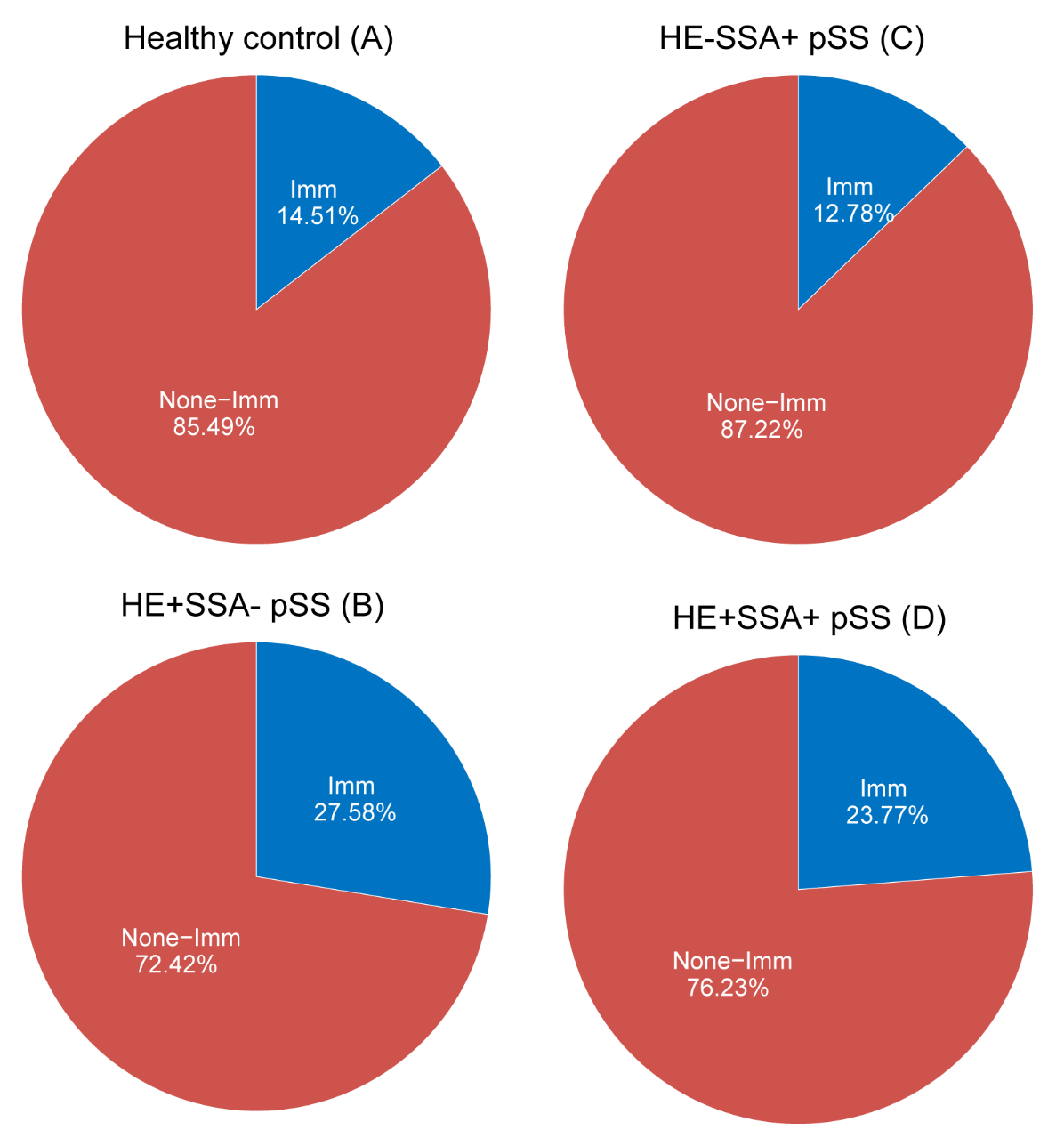
Fig. S4.** Proportion of immune and non-immune cells in different groups. Pie charts showing the percentage of immune and non-immune cells in normal control and three pSS subgroups, including HE+SSA–, HE–SSA+ and HE+SSA+ pSS patients.

**
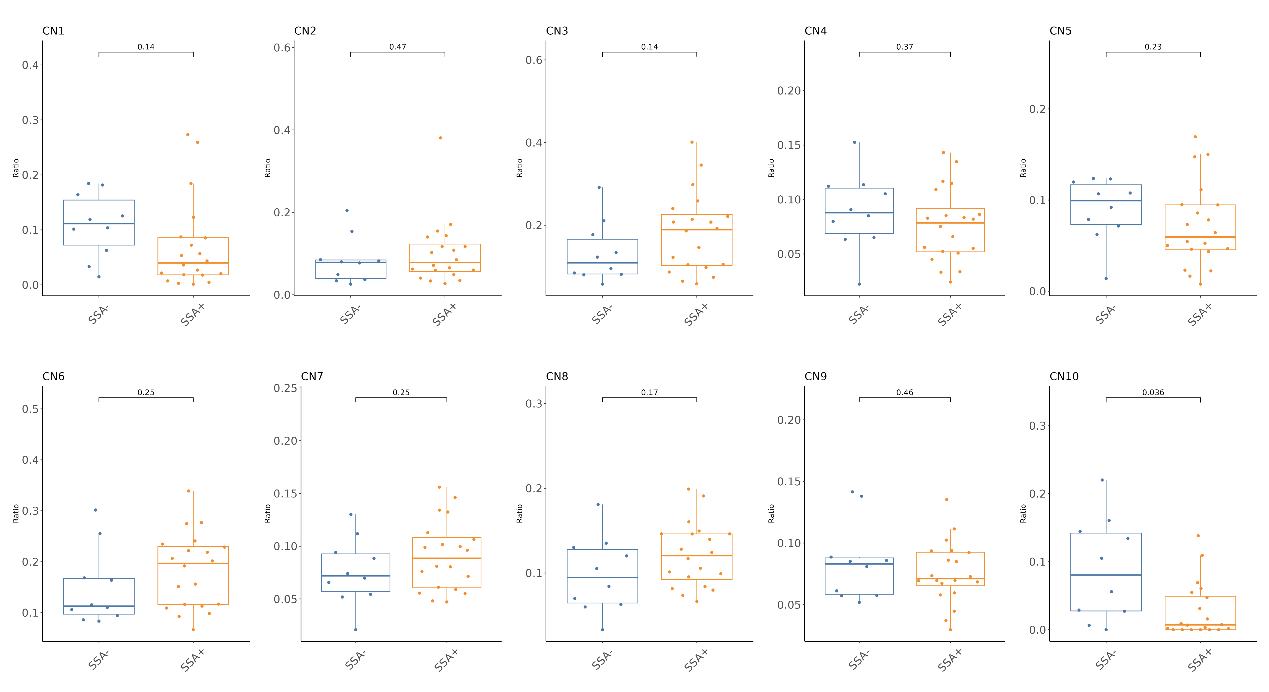
Fig. S5.** Abundance of the ten distinct cellular neighborhoods between SSA+ and SSA– pSS patients

**
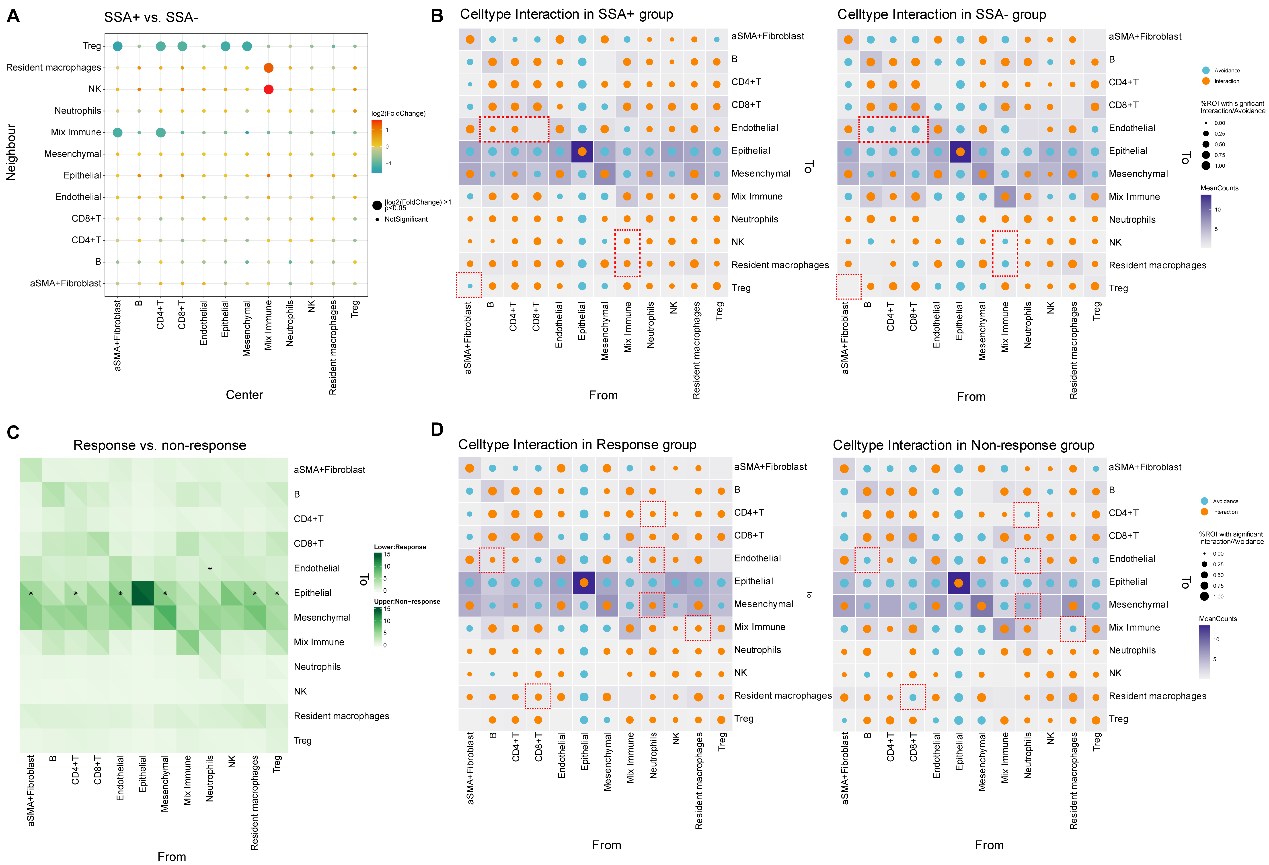
****Fig. S6.** Cell-cell spatial interactions in subgroup divided by SSA and treatment response. A, heatmap showing the differences on cell-cell communications counts between SSA+ and SSA– pSS patients, *P<0.05; B, patterns of cell-cell interactions/avoidance for cell type interaction in SSA+ and SSA– pSS patients. Orange and blue circles representing interactions pattern and avoidance pattern, respectively; A, heatmap showing the differences on cell-cell communications counts between response and non-response pSS patients, *P<0.05; B, patterns of cell-cell interactions/avoidance for cell type interaction in response and non-response pSS patients. Orange and blue circles representing interactions pattern and avoidance pattern, respectively.
