# Supplementary material for: Application of imaging mass cytometry for spatially profiling the microenvironment of salivary glands in primary Sjögren’s syndrome
Source: Cell Death Dis. 2025 May 16;16(1):392. doi: 10.1038/s41419-025-07717-7 (PMC12084299; doi:10.1038/s41419-025-07717-7)
Supplement: Supplementary file 4 — Supplementary Table 3 [file 41419_2025_7717_MOESM4_ESM.docx]

**Table S3.** The used primers in RT-qPCR.

| Name | Sequences (5’—3’) | Length (bp) |
| --- | --- | --- |
| IFN-γ (Mouse) | Forward: GCCTCGGAGTCCTCCTCTATAA | 140 |
|  | Reverse: GCGTCTCAGCCTCTTTGAGTT |  |
| TNF-α (Mouse) | Forward: ATGGCCTCCCTCTCATCAGT | 166 |
|  | Reverse: ACAAGGTACAACCCATCGGC |  |
| GZMB (Mouse) | Forward: CAGCAACAGCAAGGCGAAAA | 72 |
|  | Reverse: TGGACCTGTGGGTTGTTGAC |  |
| GAPDH (Mouse) | Forward: AGGTCGGTGTGAACGGATTTG | 123 |
|  | Reverse: TGTAGACCATGTAGTTGAGGTCA |  |
